# Synthetic Cannabinoids-Related Cardiovascular Emergencies: A Review of the Literature

**DOI:** 10.7759/cureus.41929

**Published:** 2023-07-15

**Authors:** Md Rockyb Hasan, Tanzin Tabassum, Tahsin Tabassum, Mohammed A Tanbir, Mohammed Abdelsalam, Rajesh Nambiar

**Affiliations:** 1 Internal Medicine, Texas Tech University Health Sciences Center, Amarillo, USA; 2 General Surgery, West Suffolk Hospital, Bury St. Edmunds, GBR; 3 Public Health, School of Community Health and Policy, Morgan State University, Baltimore, USA; 4 Cardiology, Texas Tech University Health Sciences Center, Amarillo, USA

**Keywords:** cardiovascular, meta-analyses, case reports, emergency management, cardiovascular complications, synthetic cannabinoids

## Abstract

Synthetic cannabinoids (SCBs) are a group of psychoactive compounds, known to cause a range of multisystem adverse events, including the cardiovascular system. The aim of this review is to provide an overview of the literature on cardiovascular emergencies associated with SCBs. A systematic search of electronic databases was conducted to identify relevant studies published between January 2010 and September 2022. Inclusion criteria were studies reporting on cardiovascular emergencies in individuals with SCB abuse. The search yielded a total of 43 studies, including case reports, case series, and meta-analyses. This review indicates that SCB abuse can lead to a range of cardiovascular emergencies, including acute coronary syndrome, arrhythmias, and hypertension. The onset of these emergencies is often sudden and may occur in previously healthy individuals. The severity of these complications can vary widely, with some cases resulting in cardiac arrest or death. Management strategies for SCB-related cardiovascular emergencies include supportive care, pharmacological interventions, and, sometimes, invasive procedures. There is no specific antidote against SCB to date. In conclusion, SCB abuse is associated with various cardiovascular emergencies, which can be life-threatening in some cases. Early recognition and management of these emergencies are critical for improving outcomes. Further research is needed to better understand the underlying mechanisms of SCB-related cardiovascular complications and to develop effective prevention and management strategies.

## Introduction and background

This article explores a hypothetical case commonly seen in a cardiology unit, highlighting the cardiac emergencies related to the use of synthetic cannabinoids (SCBs). We aim to enhance understanding of the unique challenges in diagnosing the cardiovascular effects of these substances and emphasize their significance in emergency cardiac care. A 19-year-old male named Mr. R, who had no significant past medical history, arrived at the emergency department due to cardiac chest pain and difficulty breathing. The patient's blood pressure was high at 150/90 mmHg, and he was tachycardic. Although the urine toxicology screening showed negative results, Mr. R admitted to smoking SCB 60 minutes before experiencing these symptoms. He denied having used any other illegal drugs previously. The ECG revealed ST-segment elevation in the inferior leads, without any reciprocal changes, suggesting an acute myocardial infarction. As a result, the patient underwent emergency primary coronary intervention, and the left coronary artery was found to be blocked due to thrombosis. Further laboratory studies revealed an elevated troponin I level, and dual antiplatelet therapy was initiated before transferring the patient to the cardiac intensive care unit. During the patient's hospitalization, he experienced recurrent chest pain and hypotension, necessitating vasopressor support. Subsequent ECGs showed sustained ST-segment elevation, and an echocardiogram revealed significant right ventricular dysfunction. As a result, Mr. R was diagnosed with cardiogenic shock and had an intra-aortic balloon pump placed. Gradually, the patient's condition improved, and he was successfully weaned off vasopressor support and transferred to the general medical floor. Upon discharge, he received dual antiplatelet therapy, beta-blockers, and angiotensin-converting enzyme inhibitors, and was referred to cardiac rehabilitation. The medical team also counseled him on the harmful effects of SCB.

SCBs are psychoactive designer drugs that are designed to mimic the effects of natural cannabinoids. SCBs, also known under the brand names of “Spice,” “K2,” “herbal incense,” “Cloud 9,” and “Mojo” are being widely abused [1]. Surprisingly, these have become increasingly popular among high school students [2]. Compared to natural cannabinoids, SCBs exhibit longer-lasting marijuana-like effects and lack detection by standard toxicology screens [3]. Many patients are being seen in the ER for numerous complications, including rhabdomyolysis, seizure, acute kidney injury, and arrhythmia leading to sudden cardiac arrest [4,5]. Cardiovascular emergencies are often the initial presentation of the use of SCB. There is mounting evidence of an increasing number of fatal cardiac events leading to deadly outcomes in the literature. In this review article, we will examine the mechanism of action of SCBs on the cardiovascular system, clinical presentations of cardiovascular emergencies associated with their abuse, and the management of these emergencies.

## Review

A comprehensive literature search was conducted using electronic databases such as PubMed, Embase, and Web of Science. The search included studies published between January 2010 and September 2022. The following search terms were used: "synthetic cannabinoids," "cardiovascular complications," "emergency management," "case reports," and "meta-analyses." The search strategy was developed in consultation with a medical librarian.

The following inclusion criteria were used: (a) studies reporting on cardiovascular emergencies in individuals with SCB abuse; (b) studies published in English; (c) studies published in peer-reviewed journals; (d) studies reporting on human participants; and (e) studies with a sample size of at least five participants.

The following exclusion criteria were used: (a) studies not reporting on cardiovascular emergencies in individuals with SCB abuse; (b) studies published in languages other than English; (c) studies published in non-peer-reviewed journals; (d) studies reporting on animal experiments; and (e) studies with a sample size of fewer than five participants.

Data were extracted from each included study by two independent reviewers. The data extraction included the following information: study design, sample size, patient characteristics, SCB compound used, cardiovascular outcomes, and management strategies. The extracted data were synthesized to identify common clinical presentations of cardiovascular emergencies with SCB abuse, as well as the management and prevention strategies used in different cases. The quality of the included studies was assessed using appropriate tools, such as the Cochrane risk of bias tool or the Newcastle-Ottawa Scale. Two independent reviewers assessed the quality of each study. Ethical considerations related to the use of SCB and the reporting of case studies were carefully considered. The review adhered to the ethical principles outlined in the Declaration of Helsinki and the International Committee of Medical Journal Editors (ICMJE) guidelines. The review also ensured patient confidentiality by removing any identifiable information from case reports.

SCB abuse has been becoming increasingly popular in all ages in the USA. Cardiac complications in SCB abuse are deadly and clinical suspicion with proper history taking is often helpful as detection of cases is difficult with regular toxicological screening. The mechanism of cardiac complications is still unknown but is thought to be associated with reducing blood flow from coronary vessel constriction from animal models [6]. SCBs are rapidly absorbed into the bloodstream and have longer half-lives, resulting in more rapid and durable cannabinoid effects compared to natural cannabinoids (three to five days) [7]. SCBs act as full agonists of the CB1 and CB2 cannabinoid receptors in the cardiovascular system, including the heart, blood vessels, and endothelium [8]. Activation of CB1 receptors can lead to increased heart rate and blood pressure, while activation of CB2 receptors can modulate immune function and inflammation in the cardiovascular system [9-11]. The endocannabinoid system (ECS) plays a role in many physiological processes, including pain perception, immune function, and mood regulation [12]. SCBs interact with endocannabinoids by increasing production or decreasing their breakdown. Dysregulation of the ECS has been proposed to contribute to tachycardia, fatal arrhythmia, and cardiac ischemic events [13]. SCBs can also interact with calcium channels in the heart and blood vessels, leading to a rise in intercellular calcium levels and activation of downstream signaling pathways [14]. This is how SCBs can contribute to vasoconstriction, myocardial ischemia, and arrhythmia. Additionally, SCBs can induce oxidative stress, leading to inflammation and endothelial dysfunctions in cardiovascular systems [15]. SCBs can also affect the autonomic nervous system, which contributes to cardiac symptoms and acute emergencies.

Clinical presentation of SCB abuse can range from mild to life-threatening cardiac emergencies and depends on the specific SCB compound used, dose, route of administration, and individual patient factors. Tachycardia and hypertension are commonly seen in patients who abuse SCB [16,17]. However, acute coronary syndrome, arrhythmia, and stroke are frequently reported events [18-20]. SCBs can be associated with chest pain, palpitations, dyspnea, and hypertensive emergencies with end-organ damage. Headache, dizziness, and vision changes are other notable presentations. SCBs can trigger a range of cardiac arrhythmias, including atrial fibrillation, ventricular tachycardia, and ventricular fibrillation [21]. These arrhythmias can often present with palpitation, funny feeling, syncope, or sudden cardiac arrest. Chest pain, discomfort, shortness of breath, and nausea are often related to SCB-related coronary ischemia. Chronic SCB abuse can lead to dilated cardiomyopathy and acute decompensated heart failure can be the first presentation in such cases. Takotsubo cardiomyopathy can often precipitate after SCB consumption [22].

A case report has been published describing a 16-year-old boy who suffered a myocardial infarction after smoking SCB [23]. The patient admitted that he smoked SCB with his friends. However, the duration was not mentioned. Another case report was published of a 24-year-old man who presented with ventricular fibrillation and cardiac arrest after abusing SCB [24]. In one case report, a 28-year-old man presented with acute myocardial infarction after using SCBs [25]. In both instances, the individual was actively engaging in the consumption of SCB for a minimum of one year in the company of friends. The authors suggested that SCBs may have caused vasoconstriction and increased platelet aggregation, leading to a heart attack. There is considerable evidence of ST elevated myocardial infarction following cannabis abuse [26]. Freeman et al. described a 20-year-old man who presented with an acute myocardial infarction after using SCBs. The authors suggested that the SCB may have caused vasoconstriction and increased demand of the myocardium leading to a heart attack. A 52-year-old woman with no significant past medical history was found unresponsive in cardiac arrest shortly after consuming K2-laced cigarettes [27]. Although SCB abuse is understudied, evidence suggests that marijuana may be a contributing risk factor for acute type A aortic dissection, particularly in patients with other predisposing risk factors [28]. SCB abuse was found to be associated with stress cardiomyopathy, i.e., “Takotsubo syndrome” [29]. Profound hypotension and bradycardia in the setting of SCB intoxication are reported events [30].

In a systemic review and meta-analysis, Papanti et al. analyzed the available literature on the cardiovascular effects of SCB. The authors concluded that SCB could cause cardiovascular toxicity, including hypertension, tachycardia, and arrhythmias. In a systematic review, Kousa et al. found that SCB can cause cardiovascular toxicity, including hypertension, tachycardia, and arrhythmia. Mir et al. also concluded a potential role of SCB in cardiovascular emergencies, including hypertensive crises, tachycardia, and arrhythmia [31]. A meta-analysis published on current problems in cardiology in 2018 reviewed adverse events related to cardiovascular toxicity of SCB. The meta-analysis included 51 articles and found that cardiovascular events accounted for 27.3% of all reported adverse events associated with SCB. The most reported cardiovascular events were tachycardia (55.6%), hypertension (38.9%), and chest pain (27.8%).

Management depends on a case-by-case basis and based on specific cardiac complications. General management is supportive, and no antidote is available [32]. Management of cardiovascular emergencies associated with SCB abuse should focus on stabilizing the patient's hemodynamic status, treating the underlying cardiac condition, and discontinuing using SCB [33,34]. Prevention of cardiovascular emergencies associated with SCB abuse should include education of the public and healthcare providers about the dangers of these drugs. Public health campaigns and regulatory measures can help to reduce the availability of SCBs and discourage their use. Clinicians should be aware of the potential for SCB abuse in patients who present with cardiovascular emergencies. Physicians should obtain a thorough drug history. A patient who abuses SCB should be referred to addiction treatment programs.

## Conclusions

SCB abuse can lead to life-threatening cardiovascular emergencies, including acute coronary syndrome, arrhythmias, and stroke. Clinicians should be aware of the mechanism of action of these compounds on the cardiovascular system and the potential for life-threatening complications. Teenage and young patients presenting with cardiac complications or with a history of polysubstance abuse should be asked about SCB abuse, as routine drug screen does not detect SCBs. Patients who abuse SCBs and present with cardiovascular symptoms should be evaluated promptly and treated aggressively to prevent morbidity and mortality. Further research is needed to better understand the cardiovascular effects of SCBs and to develop effective treatment and prevention strategies, as well as to establish guidelines.
